# Cholinergic Dysfunction Involvement in Chronic Cerebral Hypoperfusion-Induced Impairment of Medial Septum–dCA1 Neurocircuit in Rats

**DOI:** 10.3389/fncel.2020.586591

**Published:** 2020-09-29

**Authors:** Yi Xu, Shuai Zhang, Qiang Sun, Xu-Qiao Wang, Ya-Ni Chai, Chandan Mishra, Shah Ram Chandra, Jing Ai

**Affiliations:** Department of Pharmacology, The State-Province Key Laboratories of Biomedicine-Pharmaceutics of China, College of Pharmacy of Harbin Medical University, Harbin, China

**Keywords:** chronic cerebral hypoperfusion, septo-hippocampal neurocircuit, medial septum, acetylcholine, neurodegeneration

## Abstract

Chronic cerebral hypoperfusion (CCH) is considered a preclinical condition of mild cognitive impairment and thought to precede dementia. However, as the principal cholinergic source of hippocampus, whether the septo-hippocampal neurocircuit was impaired after CCH is still unknown. In this study, we established the CCH rat model by bilateral common carotid artery occlusion (2VO). Under anesthesia, the medial septum (MS) of rats was stimulated to evoke the field excitatory post-synaptic potential (fEPSP) in the pyramidal cell layer of dCA1. Consequently, we observed decreased amplitude of fEPSP and increased paired-pulse ratio (PPR) after 8-week CCH. After tail pinch, we also found decreased peak frequency and shortened duration of hippocampal theta rhythm in 2VO rats, indicating the dysfunction of septo-hippocampal neurocircuit. Besides, by intracerebroventricularly injecting GABAergic inhibitor (bicuculline) and cholinergic inhibitors (scopolamine and mecamylamine), we found that CCH impaired both the pre-synaptic cholinergic release and the post-synaptic nAChR function in MS–dCA1 circuits. These results gave an insight into the role of CCH in the impairment of cholinergic MS–dCA1 neurocircuits. These findings may provide a new idea about the CCH-induced neurodegenerative changes.

## Introduction

The integrity of basal forebrain–hippocampal neurocircuit is essential for the regulation of spatial and episodic memory (Ballinger et al., 2016). By performing high-resolution magnetic resonance imaging, Taylor W. Schmitz’s team has found that the degeneration of the basal frontal brain occurs before cognitive impairment and pathological changes in the entorhinal layer of patients with Alzheimer’s disease (AD; Schmitz and Nathan Spreng, 2016). Furthermore, using anterograde and retrograde labeling techniques, it has been found that, in 5XFAD mice, the degeneration of nerve fibers in the basal forebrain–hippocampus loop is earlier than the death of neurons (Kim et al., 2019). These studies suggest that impairment of the basal forebrain–hippocampal neurocircuit may be an early indicator of AD occurrence. However, inducements and mechanisms are still not fully elucidated.

Chronic cerebral hypoperfusion (CCH) is considered a common cause of sporadic AD and vascular dementia (VaD; Cole and Vassar, 2009; Gorelick et al., 2011; Honjo et al., 2012) and a subclinical state of moderate cognitive impairment (Maalikjy Akkawi et al., 2005; Ruitenberg et al., 2005; Gorelick et al., 2011). Many previous studies have reported that CCH induces cognitive decline by trigging a variety of hippocampal pathologies, including β-amyloid deposition (Ai et al., 2013; ElAli et al., 2013), tau phosphorylation (Sun et al., 2015; Liu et al., 2016; Raz et al., 2019), neuronal death (Chen et al., 2017), and even impairment of hippocampal pre-synaptic plasticity (Yan et al., 2020). However, it is still unknown whether and how CCH initiates the impairment of basal forebrain–hippocampal neurocircuit.

It has been well studied that the medial septum (MS) within basal forebrain is one of the brain regions that closely interact with hippocampal CA1. This interaction is a precise negative feedback adjustment system [septo-hippo-septal loop (SHSL)], which adjusts the CA1 network excitability to different behavioral states (Müller and Remy, 2018). Although SHSL is controlled by the synergy of cholinergic, glutamatergic, and GABAergic neurons together, convincing evidence has demonstrated that cholinergic circuit in SHSL places a center stage for normal executive and mnemonic functioning, and the loss of cholinergic signaling is inextricably linked with cognitive decline (Dutar et al., 1995; Teles-Grilo Ruivo and Mellor, 2013; Ballinger et al., 2016; Dannenberg et al., 2017; Solari and Hangya, 2018). Recently, Li et al. (2019) reported that CCH impairs the hippocampal theta synchrony between CA3 Schaffer collaterals and CA1 areas in 2-week 2VO rats. However, whether 8-week CCH rats display an impaired function of SHSL *in vivo* and whether the cholinergic dysfunction is involved in this process are still unclear.

In this study, we employed the electrophysiological methods and found that CCH impaired the cholinergic circuits from MS to the pyramidal cell layer of dCA1. This study is the first to elucidate the impairment of MS–dCA1 neurocircuit in CCH rat models; and it will provide an important reference for the future mechanism study and drug research.

## Materials and Methods

### Animals

Male Sprague–Dawley (SD) rats (weight 220–260 g, obtained from the Animal Center of the Second Affiliated Hospital of Harbin Medical University, Harbin, Heilongjiang Province, China) were housed at 23 ± 1°C with 55 ± 5% of humidity and maintained on 12-h dark/light artificial cycle (lights on at 7:00 A.M.) with food and water available *ad libitum*. Rats for creating permanent, bilateral common carotid artery occlusion (2VO) were anesthetized with chloral hydrate (300 mg/kg) by intraperitoneal injection and maintained by administrating 0.5–1.0% isoflurane. The depth of anesthesia was monitored by detecting reflexes, heart rate, and respiratory rate. All animal procedures were approved by the Institutional Animal Care and Use Committee at Harbin Medical University and the Institute of Laboratory Animal Science of China. All procedures were conformed to the Directive 2010/63/EU of the European Parliament.

### Permanent Bilateral Common Carotid Artery Occlusion

The method used for the preparation of 2VO rats has been described in previous studies (Ai et al., 2013). Briefly, after anesthesia, the bilateral common carotid arteries of rats were exposed *via* a midline ventral incision, carefully separated from the vagal nerves, and then permanently ligated with 5-0 silk suture. After the surgical procedures, all the anterior cervical muscles were returned to their original locations. To avoid potential postoperative infection, the wounds were washed with 20 mg/ml of gentamycin sulfate solution before being sutured. The rats were then allowed to recover from anesthesia before being returned to their cages.

### Surgical Procedures and Placement of Electrodes

Under urethane anesthesia (1.2 g/kg of body weight, supplemental doses of 0.2–0.8 g/kg as needed), the rats were mounted on the stereotaxic frame apparatus (DW-2000, Chengdu Taimeng Software Company Limited, China) for the insertion of electrodes. For local anesthesia, procaine hydrochloride (1%) was injected subcutaneously into the tissue around the incision before surgery. A heating pad was placed under the rat to maintain the body temperature of mice at 37°C. After holes were drilled into the skull, a bipolar stimulating electrode (stainless steel, tip separation 0.5 mm) was implanted for stimulating the MS [anterior–posterior (AP), 0.6 mm; medio-lateral (ML), 0.1 mm; dorso-ventral (DV), 6.0 mm]. In order to avoid the changes in the impedance of stimulating electrodes, each stimulating electrode was prepared with two pieces of 10-cm stainless steel with 0.25-mm diameter and carefully cleaned before and after usage. The recording electrode filled with 3 mol/L of NaCl was placed in the pyramidal cell layer of the dorsal CA1 (AP, −3.8 mm; ML, 2.4 mm; DV, 2.7 mm). The depths of the stimulating and recording electrodes were adjusted to obtain the maximal responses (Jedlicka et al., 2009; Yang et al., 2011).

### Electrophysiological Recording and Analysis

After a 30-min recovery from electrode implantation, MS was stimulated by constant current pules with 30-s interval (0.1-ms duration) from BL-420S stimulus generator (Chengdu Taimeng Software Company Limited, China). The field excitatory post-synaptic potential (fEPSP) recorded in dCA1 was amplified by a ME-1 preamplifier (Chengdu Taimeng Software Company Limited, China); and the signals were then filtered (1 Hz–1 kHz), digitized (20 kHz), and recorded by the software of BL-420S. In the recording system, baseline was normalized to 0 mV, and the extracellularly recorded cation influx (excitatory response) is defined as up-word waveform. To establish the input–output (I/O) curves, electric stimulation with intensity ranged from 1 to 20 V were applied to MS. The amplitude and slope of post-synaptic potentials were expressed as the average of three responses under each stimulation intensity. Based on the I/O curve, the stimulus intensity corresponding to the 50% maximum amplitude of post-synaptic potential was used for recording the paired-pulse ratio (PPR). The inter-stimulus interval was set between 20 and 70 ms. The PPR is calculated by dividing the second pulse amplitude by the first pulse amplitude. The latency of post-synaptic potential was determined by the time between the arrival of a pre-synaptic action and the onset of post-synaptic response. The time to peak of post-synaptic potential was the time interval from the onset of this measured potential to the peak amplitude. The half-width of post-synaptic potential is the width (duration) between half-maximal peak.

### Local Field Potential

The local field potentials (LFPs) of dCA1 were recorded as described in previous studies (Varga et al., 2008; Liu et al., 2013). Briefly, rats were anesthetized by intraperitoneally injected 20% urethane solution and placed on a stereotaxic frame. The animals were maintained at a level of anesthesia at which spontaneous theta rhythm was not presented but could be elicited by a tail pinch. Monopolar tungsten electrode was implanted into the pyramidal cell layer of the dorsal CA1 (AP, −3.8 mm; ML, 2.4 mm; DV, 2.7 mm) to record hippocampal LFP. The reference electrode was placed on the skull 2 mm away from the recording electrode, and the ground electrode clamped the skin on both sides of the brain. The LFP of dCA1 was amplified by a ME-1 preamplifier (Chengdu Taimeng Software Company Limited, China); and then the signals were filtered (0–20 Hz) and recorded by the software of BL-420S. After the LFP waveform became stable, the basic spontaneous LFP was recorded for 2 min. Subsequently, the vicinity of tail base was gently pinched with a plastic clamp for 1 min to induce theta rhythm.

### Intracerebroventricular Drug Infusions

At the beginning of each recording, a hole was drilled above the lateral cerebral ventricle of the skull (AP, 1.0 mm; ML, 1.5 mm; DV, 4.0 mm). Then, a 5-μl Hamilton syringe with a 33-gauge tip needle (Hamilton, Bonaduz, Switzerland) containing bicuculline (1 μg/rat), scopolamine (10.8 μg/rat), or mecamylamine (10 μg/rat) was inserted into the drilled lateral cerebral ventricle of rats during the electrophysiological recording process. The drugs (2 μl) were infused at a rate of 0.2 μl/min. After 10 min of drug administration, the electrophysiological recording was continued, and the needle was indwelling until the whole recording was finished. Bicuculline, scopolamine, and mecamylamine were purchased from Aladdin (Shanghai Aladdin Bio-chem Technology Company Limited, China). Bicuculline was dissolved in dimethyl sulfoxide (DMSO). Scopolamine and mecamylamine were dissolved in saline. All drugs were freshly prepared before usage.

### Western Blotting

Total hippocampal proteins were extracted, and the protein content was determined by the BCA Protein Assay Kit using bovine serum albumin as an internal standard. Protein samples fractionated on a 10% sodium dodecyl sulfate–polyacrylamide gel electrophoresis (SDS–PAGE) were transferred onto nitrocellulose membranes and incubated with primary antibody against ChAT (Catalog. #297013, 1:1,000, Synaptic Systems) and β-actin (Catalog. #8432, 1:1,000, Santa Cruz). The membranes were then incubated with fluorescent secondary antibody. The protein bands were captured using the Odyssey Infrared Imaging System (LI-COR Biosciences, Lincoln, NE, USA) and quantified using the Odyssey version 3.0 software. β-Actin was used as an internal control for protein inputs.

### Assessment of Acetylcholine Concentration

The hippocampal sample used for ELISA analysis was prepared as described in the instructions. Briefly, frozen tissue samples from the dorsal hippocampus were homogenized in phosphate-buffered saline (PBS; pH 7.4) at 4°C. The extracts were centrifuged at 3,000 rpm for approximately 20 min; and the supernatants were collected carefully. After extraction, the acetylcholine levels were measured immediately using the rat acetylcholine (Ach) ELISA Kit (Catalog. #MBS774123, MyBioSource) according to the instruction of the manufacturer. Finally, according to the standard sample’s concentrations and the corresponding optical density (OD) values, the linear regression equation of the standard curve was fitted. Based on this equation, the Ach concentrations of test samples were calculated.

### Immunofluorescence Detection

After the electrophysiological and LFP recording, rats were perfused transcardially with 4% buffered paraformaldehyde (PFA). The brains were then removed, dehydrated, and frozen in OCT; and 30-μm brain slices were mounted on glass slides. Subsequently, the brain slices were incubated with DAPI (C1005, 1:100, Beyotime) for 30 min and then washed and covered with mounting medium. Finally, brain slices were observed by brightfield microscopy using a ×5 objective on a Zeiss Axio Scope A1 microscope.

### Statistical Analysis

Data were described as mean ± SEM. The power spectrums of LFPs were analyzed by Matlab 7.0. The two-tailed Student’s *t*-test was applied for comparisons between the two groups; *P* < 0.05 was considered statistically significant. SAS 9.1 software (serial number: 989155, Institute Incorporation) was used for all statistical analyses.

## Results

### Chronic Cerebral Hypoperfusion Impairs the Basic Electrophysiological Properties of Medial Septum–dCA1 Neurocircuit

**Figure 1:**
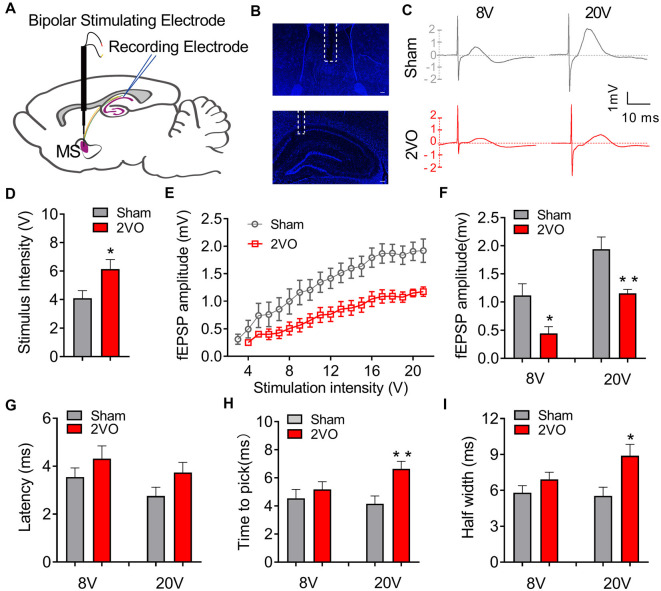
Chronic cerebral hypoperfusion (CCH) impairs the basic neurotransmission process of medial septum (MS)–dCA1 neurocircuit. **(A)** Schematic diagram showing the placement of the stimulating (MS) and recording (dCA1) electrodes during *in vivo* electrophysiological experiment. **(B)** Immunostaining of brain slices; the broken white lines show the position of stimulating and recording electrodes trace. Scale bar = 200 μm. **(C)** Sample MS–dCA1 field excitatory post-synaptic potential (fEPSP) traces in the sham and bilateral common carotid artery occlusion (2VO) rats with 8- and 20-V stimulations. **(D)** Differences of the lowest stimulus intensity needed for evoking a measurable response of MS–dCA1 circuit. *n* = 10 for sham rats, *n* = 7 for 2VO rats. **(E)** Differences of the fEPSP amplitude between the sham and 2VO rats. *n* = 10 for sham rats, *n* = 7 for 2VO rats. **(F)** Comparison of input–output curves between sham and 2VO rats. *n* = 8 for sham rats, *n* = 7 for 2VO rats. **(G–I)** Comparison of the latency, time to peak, and half width of MS–dCA1 fEPSP in sham and 2VO rats at 8- and 20-V stimulations. *n* = 10 for sham rats, *n* = 7 for 2VO rats. *P < 0.05 vs. sham rats, **P < 0.01 vs. sham rats.

To investigate whether CCH could induce the dysfunction of the septo-hippocampal neurocircuit, we first evaluated the basic electrophysiological properties of the MS–dCA1 neurocircuit *in vivo*. A stimulating electrode was implanted in the rat MS, and the post-synaptic potential was recorded in the pyramidal cell layer of dCA1 region (Figures 1A–C; Li et al., 2019). First of all, the responsiveness of MS–dCA1 neurocircuit in 2VO rats was evaluated. As shown in Figure 1D, compared with sham rats, dramatically higher stimulation intensity was required to elicit the electrical activity of MS–dCA1 circuits in 2VO rats (4.1 ± 0.53 vs. 6.14 ± 0.67 V, *P* = 0.0283). It suggests that the responsiveness of MS–dCA1 neurocircuit was impaired. Next, we wanted to explore the potential causes through analyzing various electrophysiological properties of MS–dCA1 circuits. We administrated a 1-V-step increased stimulation to MS-CA1 circuits and found that the fEPSP amplitudes of sham and 2VO rats increased with the enhancement of stimulus intensity (Figure 1E). However, the fEPSP amplitude in 2VO rats was significantly lower than that in sham rats (8 V, 1.12 ± 0.21 vs. 0.45 ± 0.12 mV, *P* = 0.0238; 20 V, 1.94 ± 0.22 vs. 1.15 ± 0.07 mV, *P* = 0.0078; Figure 1F). These results suggest that the basic neurotransmission process of MS–dCA1 circuits was impaired in 2VO rats. To further understand the impaired electrophysiological activity of MS–dCA1 circuits in 2VO rats, we continued to evaluate the detailed characteristics of fEPSP waveform, including latency, time to peak, and half width. Latency, the time interval between the arrival of a pre-synaptic action potential and the onset of a post-synaptic response, can be used to infer the kinetics of quantal transmitter release (Lin and Faber, 2002). As shown in Figure 1G, under 8- or 20-V electric stimulation, there were no differences of fEPSP latencies between 2VO and sham rats, indicating the intact kinetics of transmitter release and transmission speed of MS–dCA1 circuits in 2VO rats. Time to peak and half width reveal the response speed of activated receptors go from low to high ionic permeability (Lin and Faber, 2002). Here, we found that the time to peak (4.16 ± 0.55 vs. 6.65 ± 0.53 ms, *P* = 0.0057; Figure 1H) and half width (5.54 ± 0.72 vs. 8.89 ± 0.95 ms, *P* = 0.0137; Figure 1I) of MS–dCA1 circuit of 2VO rats were extended under 20-V but not 8-V electric stimulation. These analyses of fEPSP waveform properties suggested that 8-week CCH weakened the response of receptors following high intensity of stimulation in MS–dCA1 circuits. Collectively, CCH impaired the connection between the MS and the dCA1 pyramidal cells, as well as the responsive ability of dCA1 to high-intensity stimuli but did not impair the kinetics of transmitter release and transmission speed of MS–dCA1 circuit.

**Figure 2:**
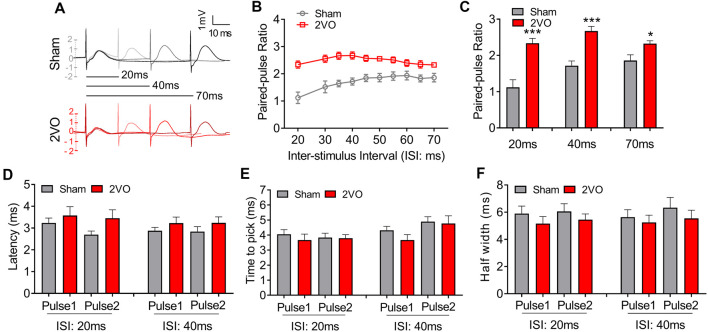
CCH impairs the paired-pulse facilitation (PPF) of medial septum (MS)–dCA1 neurocircuit. **(A)** Sample MS–dCA1 fEPSP traces during paired pulse with 20, 40, and 70 ms of inter-stimulus intervals. **(B)** Scatter plot summarizing the paired-pulse ratio (PPR) curve in sham and bilateral common carotid artery occlusion (2VO) rats. *n* = 12 for sham rats, *n* = 8 for 2VO rats. **(C)** Differences of the PPR between the sham and 2VO rats with 20, 40, and 70 ms of inter-stimulus interval stimulated by 8 V. Left insertion: sample fEPSP traces of the sham and 2VO rats. *n* = 12 for sham rats, *n* = 8 for 2VO rats. **(D–F)** Comparison of the latency, time to peak, and half width of MS–dCA1 fEPSP during PPF in sham and 2VO rats. *n* = 12 for sham rats, *n* = 8 for 2VO rats. *P < 0.05 vs. sham rats, ***P < 0.001 vs. sham rats.

To further verify the pre-synaptic properties, we applied paired pulse with 8-V stimulus intensity to the MS–dCA1 circuit. In central synapses, paired-pulse facilitation (PPF) is defined as follows: the second stimulus evokes a larger response than the first when two stimuli are delivered within hundreds of milliseconds to seconds of each other (Regehr, 2012). An increase in PPR during PPF induction indicates a decrease in the probability of pre-synaptic neurotransmitter release (Deng et al., 2020). Thus, we applied paired pulses with 20- to 70-ms interval to MS to induce PPF (Figure 2A) and found significantly higher PPR in 2VO rats, with the greatest effects at the shortest inter-stimulus interval (20 ms, 1.12 ± 0.21 vs. 2.34 ± 0.13, *P* = 0.0003; 40 ms, 1.72 ± 0.13 vs. 2.67 ± 0.13, *P* < 0.0001; 70 ms, 1.86 ± 0.16 vs. 2.32 ± 0.08, *P* = 0.0346; Figures 2B,C) than sham rats. The same to those at basal neurotransmission condition, the latency (Figure 2D), time to peak (Figure 2E), and half width (Figure 2F) of MS–dCA1 fEPSP were not changed between sham and 2VO rats. Thus, 8-week CCH impairs the pre-synaptic function of MS–dCA1 neurocircuit.

### Chronic Cerebral Hypoperfusion Damages the Theta Rhythm of dCA1

**Figure 3:**
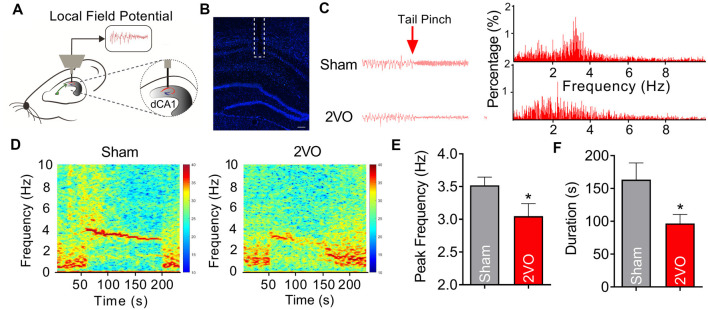
CCH damages the theta rhythmogenesis of dCA1. **(A)** Schematic diagram showing the recording method of theta rhythm in dCA1. **(B)** Immunostaining of brain slice; the broken white lines show the position of local field potential (LFP) recording electrode trace. Scale bar = 200 μm. **(C)** Comparison of the dCA1 LFP in sham and bilateral common carotid artery occlusion (2VO) rats. Left panel: sample dCA1 LFP waveforms. Right panel: frequency spectrum analysis of the left waveforms. **(D)** Heatmaps showing the frequency spectrum characteristics of the dCA1 LFP in sham and 2VO rats. **(E,F)** Comparison of the peak frequency and duration of theta rhythm in sham and 2VO rats. *n* = 12 for sham rats, *n* = 9 for 2VO rats, *P < 0.05 vs. sham rats.

MS, which is reciprocally connected with hippocampus, is thought to be the rhythm generator of theta (Hangya et al., 2009; Unal et al., 2015). Theta oscillations are LFP, which reflect rhythmic changes of the synaptic inputs to the hippocampus (Bragin et al., 1995). In order to further verify the phenomenon of the impaired fEPSP of MS–dCA1 in anesthetized 2VO rats, we recorded hippocampal LFP in the pyramidal cell layer of dCA1 region in sham and 2VO rats (Hangya et al., 2009; Figures 3A,B). After a 1-min tail pinch, the hippocampal LFP shifted from delta rhythm to theta rhythm (Figures 3C,D). However, we found that the peak theta frequency of 2VO rats (3.05 ± 0.19 Hz) was significantly lower than that of sham rats (3.52 ± 0.12 Hz, *P* = 0.0444; Figure 3E). Besides, compared with sham rats (163.45 ± 25.44 s), 2VO rats (96.67 ± 13.83 s, *P* = 0.0443) showed a significantly shortened duration of hippocampal theta rhythm after tail pinch (Figure 3F). These results suggest that the hippocampal theta rhythmogenesis was impaired in 2VO rats; and this phenomenon further demonstrated that the MS–dCA1 neurocircuit dysfunction occurred after 8 weeks of CCH.

### Chronic Cerebral Hypoperfusion Induces Diminished Pre-synaptic Cholinergic Function of Medial Septum–dCA1 Circuit

**Figure 4:**
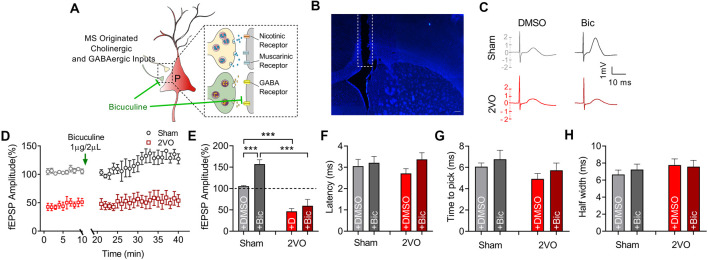
CCH impairs the basic neurotransmission of cholinergic medial septum (MS)–dCA1 neurocircuit. **(A)** Schematic diagram showing the intracerebroventricular injection of bicuculline and drug action in medial septum (MS)–dCA1 neurocircuit. **(B)** Immunostaining of brain slices; the broken white lines show track of needle used for intraventricular drug administration. Scale bar = 200 μm. **(C)** Sample MS–dCA1 fEPSP traces after administration of bicuculline. **(D)** Comparison of normalized fEPSP after the injection of bicuculline between sham and bilateral common carotid artery occlusion (2VO) rats. *n* = 6 for sham rats, *n* = 6 for 2VO rats. **(E)** Column graph shows the difference of normalized fEPSP amplitude before and after the injection of bicuculline in sham and 2VO rats. Upper insertion: sample fEPSP traces before and after the injection of bicuculline in sham and 2VO rats. *n* = 6 for sham rats, *n* = 6 for 2VO rats. **(F–H)** Column graph shows the influence of bicuculline on the fEPSP latency, time to peak, and half width. *n* = 6 for sham rats, *n* = 6 for 2VO rats. ***P < 0.001 vs. sham rats.

MS–dCA1 neurocircuit contains cholinergic and GABAergic projections. Functionally, the cholinergic and GABAergic projections mainly contribute to the regulation of hippocampal function and the post-synaptic potentials of septo-hippocampal pathway (Müller and Remy, 2018). When MS was stimulated, we could record a mixed excitatory and inhibitory potential in dCA1. But at this time, the inhibitory response neutralized part of the excitatory potential. Therefore, we intracerebroventricularly injected bicuculline (1 μg/2 μl) to block the GABAergic function and to separately evaluate the excitatory component of MS–dCA1 circuit (Figures 4A,B). After 10 min of bicuculline administration, we stimulated the MS and observed a 57.1% increase of fEPEP amplitude in dCA1 of sham rats. This 57.1% increase of fEPEP amplitude is the response neutralized by fIPSP when bicuculline did not exit. After the mixed response was normalized to 100%, we calculated that the bicuculline-sensitive potential fIPSP accounted for 26.7% [0.571/(0.571 + 1.571)] of the total MS–dCA1 post-synaptic potential, which was close to the percentage in previous studies (Sun et al., 2014). The phenomenon indicated that the GABAergic component of MS–dCA1 circuit was totally inhibited. Subsequently, we observed a significantly decreased amplitude of cholinergic fEPSP in 2VO rats (157.08 ± 10.09% vs. 59.36 ± 15.25%, *P* = 0.0003; Figures 4C–E). Besides, bicuculline administration induced a dramatic increase of MS–dCA1 fEPSP in sham rats (105.41 ± 1.51% vs. 157.08 ± 10.09%, *P* = 0.0005), while this phenomenon was absent in 2VO rats (46.58 ± 6.45% vs. 59.36 ± 15.25%, *P* = 0.4581). These results indicated that the GABAergic neurotransmission of MS–dCA1 circuit was impaired by CCH (Figures 4C–E). However, there were no differences in latency (Figure 4F), time to peak (Figure 4G), and half width (Figure 4H) of the cholinergic fEPSP between sham and 2VO rats, suggesting that CCH impairs the cholinergic function between MS and dCA1 pyramidal cells but did not affect the kinetics of transmitter release and transmission speed of MS–dCA1 circuit.

**Figure 5:**
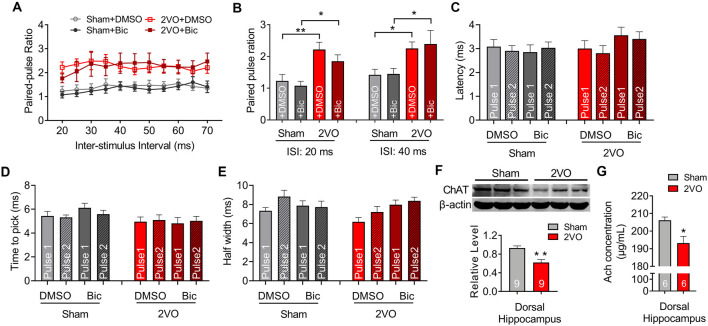
CCH impairs the pre-synaptic function of cholinergic and GABAergic medial septum (MS)–dCA1 neurocircuit. **(A)** Scatter plot summarizing the PPR curve before and after the injection of bicuculline in sham and bilateral common carotid artery occlusion (2VO) rats. *n* = 6 for sham rats, *n* = 6 for 2VO rats. **(B)** Column graph shows the differences of the PPR before and after the injection of bicuculline in sham and 2VO rats with 20 and 40 ms of inter-stimulus intervals. *n* = 6 for sham rats, *n* = 6 for 2VO rats. **(C–E)** Column graph shows the influence of bicuculline on the fEPSP latency, time to peak, and half width during PPF. *n* = 6 for sham rats, *n* = 6 for 2VO rats. **(F)** Western blot analysis with the dorsal hippocampus in sham and 2VO rats. Top: representative immunoblotting images of ChAT and β-actin. Bottom: the digital data of the immunoblotting analysis. *n* = 9 for sham rats, *n* = 9 for 2VO rats. **(G)** Comparison of the dCA1 Ach concentrations in sham and 2VO rats. *n* = 6 for sham rats, *n* = 6 for 2VO rats. *P < 0.05 vs. sham rats, **P < 0.01 vs. sham rats.

However, from the diminished cholinergic fEPSP amplitude, we still cannot determine whether the reduced release of the pre-synaptic transmitter or the activation of the post-synaptic receptor contributed to the impaired septo-hippocampal connectivity (Lin and Faber, 2002; Zamudio-Bulcock and Valenzuela, 2011). Therefore, the paired-pulse stimulating protocol was then applied to MS–dCA1 circuit to evaluate the pre-synaptic cholinergic function. As predicted, compared with that of sham rats (1.08 ± 0.14), the PPR of MS–dCA1 circuit in 2VO rats (1.85 ± 0.2, *P* = 0.0103) was significantly increased, indicating the decrease of cholinergic release probability (Figures 5A,B). The latency (Figure 5C), time to peak (Figure 5D), and half width (Figure 5E) of the cholinergic fEPSP were similar among all groups. Furthermore, we found the expression level of dorsal hippocampal ChAT was dramatically decreased in 2VO rats (0.93 ± 0.05 vs. 0.62 ± 0.06, *P* = 0.0011; Figure 5F), which further verified the impairment of hippocampal cholinergic inputs. As a supplementation of the diminished cholinergic release, Ach concentration of dorsal hippocampus in 2VO rats was significantly lower than that in sham rats (206.13 ± 1.86 vs. 193.18 ± 3.81 μg/ml, *P* = 0.0121; Figure 5G). Therefore, these results collectively indicated that the pre-synaptic cholinergic function of MS–dCA1 circuit was impaired in 8-week CCH rats.

### Chronic Cerebral Hypoperfusion Induces Cholinergic Receptor Dysfunction of Medial Septum–dCA1 Circuit

**Figure 6:**
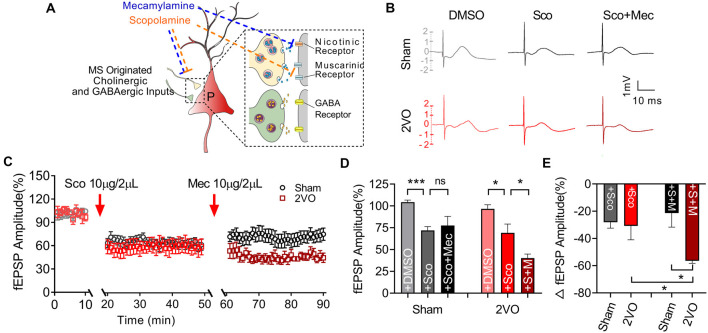
Medial septum (MS)–dCA1 neurocircuit in CCH rat was more sensitive to cholinergic inhibition. **(A)** Schematic diagram showing the intracerebroventricular injection of scopolamine and mecamylamine. **(B)** Sample MS–dCA1 fEPSP traces after administration of scopolamine and mecamylamine. **(C)** Comparison of normalized fEPSP after the administration of cholinergic inhibitors in sham and bilateral common carotid artery occlusion (2VO) rats. *n* = 8 for sham rats, *n* = 7 for 2VO rats. **(D,E)** Column graph shows the difference of normalized and changed fEPSP amplitude before and after the injection of scopolamine and mecamylamine in sham and 2VO rats. Upper insertion: sample fEPSP traces before and after the injection of scopolamine and mecamylamine in sham and 2VO rats. *n* = 8 for sham rats, *n* = 7 for 2VO rats. *P < 0.05 vs. sham rats, ***P < 0.001 vs. sham rats. ns, no statistical significance.

Subsequently, we would like to further clarify whether the function of post-synaptic cholinergic receptors in the MS–dCA1 circuit was impaired after CCH. Cholinergic receptors include muscarinic acetylcholine receptor (mAChR) and nicotinic acetylcholine receptors (nAChR). mAChRs were identified as five subtypes, termed M1–5. These receptors are expressed in both the hippocampal pyramidal neurons and interneurons (Levey et al., 1995; Bell et al., 2013; Alger et al., 2014). M1, M3, and M5 are enriched in pyramidal neurons (Vilaró et al., 1990; Levey et al., 1995) and lead to Ca^2+^ influx and activate intracellular signaling cascades by coupling to Gq proteins (Wess, 2003), while M2 and M4 are expressed mostly in nonpyramidal neurons (Levey et al., 1995) and coupled to G_i/o_ proteins to inhibit hippocampal inhibitory interneurons (Wess, 2003). Therefore, a mild concentration of mAChR (10 μg/2 μl of scopolamine) blocker was used to evaluate mAChR function in MS–dCA1 circuits (Liu et al., 2018; Figure 6A). Based on the decreased cholinergic fEPSP amplitude shown in Figure 4, and the previously reported disrupted central muscarinic system in AD (Scarr, 2012), we conjectured that the application of muscarinic inhibitor would lead to a bigger drop of the MS–dCA1 fEPSP in 2VO rats. However, to our surprise, the application of 10 μg/2 μl of scopolamine led to the same decreases of the MS–dCA1 fEPSPs in sham and 2VO rats (71.94 ± 4.16% vs. 69.14 ± 3.65%, *P* = 0.3088; Figures 6B–E). This result suggested that CCH did not significantly influence the mAChR function.

Except for mAChR, hippocampal nAChR is also reported to express on glutamatergic pre-synaptic neurons and to enhance the release of glutamate (Cheng and Yakel, 2014; Dannenberg et al., 2017). Pharmacological experiments have shown that muscarinic receptor antagonism with scopolamine results in deficits in working and declarative memory. Simultaneous antagonism of both muscarinic and nicotinic receptors produced greater deficits than muscarinic blockade alone, while nicotinic antagonism alone with mecamylamine did not show similar deficits (Ellis et al., 2006). Hence, a non-selective nAChR blocker, mecamylamine (10 μg/2 μl; Papke et al., 2001) was used to evaluate the effect of CCH on nAChR function based on scopolamine inhibition action on mAChR (Figure 6A). After the application of nicotinic inhibitors, we found a further decrease of the MS–dCA1 fEPSP in 2VO rats (69.14 ± 3.65% vs. 40.29 ± 3.45%, *P* = 0.0317), while the MS–dCA1 fEPSP did not change in sham rats (71.94 ± 4.16% vs. 77.59 ± 3.83%, *P* = 0.5959; Figures 6B–E). The internal mechanism of this phenomenon may be that the post-synaptic receptors cannot be saturated by one time of transmitter release at normal conditions (Frerking and Wilson, 1996; Edwards, 2007). This is to say, in sham rats, a single release of Ach cannot completely occupy the post-synaptic nAChR, so mild inhibition of nAChR cannot affect the post-synaptic response. However, since the reserve of hippocampal nAChR was impaired in 2VO rats, a low-level mecamylamine induced a significant decrease of fEPSP in MS–dCA1 circuit. This increased sensitivity to mecamylamine suggested that the nAChR function of the MS–dCA1 neurocircuit was decreased in 2VO rats.

## Discussion

CCH is considered to be the preclinical stage of mild cognitive impairment (Du et al., 2017); and it has already been proved to participate in the β-amyloid deposition, tau phosphorylation, neuronal death, impairment of hippocampal pre-synaptic plasticity, and theta oscillation in rats (Ai et al., 2013; Sun et al., 2015; Liu et al., 2016; Chen et al., 2017; Li et al., 2019; Yan et al., 2020). However, as the largest cholinergic nucleus and the theta generator of brain, whether the function of MS could be impaired by CCH is still unelucidated. Here, we are the first to focus on the influence of CCH on septo-hippocampal neurocircuit.

*In vivo*, an important function of septo-hippocampal pathway is adjusting the CA1 network excitability to different behavioral states (Müller and Remy, 2018). To evaluate the effect of CCH on septo-hippocampal function, we established the 2VO rat models and recorded the electrophysiological properties of pyramidal cell layer in dCA1 through stimulating MS. By recording and analyzing the I/O curves, we found a significantly decreased post-synaptic potential amplitude in the MS–dCA1 circuit of 2VO rats without affecting the kinetics of transmitter release and post-synaptic response process. Thereafter, PPR recording revealed that CCH impaired pre-synaptic neurotransmitter release. Since the normal function of cholinergic and GABAergic MS–dCA1 circuit is the basis for maintaining the hippocampal theta oscillations (Zhang et al., 2010; Rubio et al., 2012; Vandecasteele et al., 2014; Vega-Flores et al., 2014), changes of hippocampal theta rhythm would be the best alternative index to reflex the function of the MS–dCA1 circuit. Previous studies have reported that hippocampal theta rhythm can be recorded both in alert behaving and urethane-anesthetized animals *in vivo* or in brain slices *in vitro* (Varga et al., 2008; Liu et al., 2013; Hernández-Pérez et al., 2016; Li et al., 2019), and the hippocampal theta rhythm shifts to 3–4 Hz in urethane-anesthetized rats (Varga et al., 2008; Liu et al., 2013). In the present study, we selected LFP recording in urethane-anesthetized animals following tail pinch to assess the theta rhythm to keep consistent with the recording method of fEPSP; and we observed that hippocampal theta rhythm significantly decreased as indicated by decreased peak frequency and duration. The result was similar to that reported previously *in vitro* in 2VO rats for 3 weeks (Li et al., 2019). All these results soundly demonstrated that 8-week CCH impaired the septo-hippocampal function.

Previous morphological evidences have shown that the projection from MS to the pyramidal cells of dCA1 mainly composed of cholinergic and GABAergic components (Sun et al., 2014); and both the cholinergic and GABAergic functions were reported reduced in AD model (Loreth et al., 2012; Schmid et al., 2016). Here, to illustrate whether CCH could affect cholinergic function, cholinergic component of MS–dCA1 circuit were extracted by intracerebroventricularly injecting bicuculline. We found that, compared with that of sham rats, the amplitude of cholinergic fEPSP in 2VO rats was significantly decreased, suggesting impaired cholinergic transmission.

Interestingly, although CCH diminished fEPSP of MS–dCA1 circuit, it did not affect the kinetics of transmitter release and post-synaptic response process. This suggested that decreased presynaptic neurotransmitter release or declined postsynaptic activated receptor might be involved in CCH-induced physiological remodeling process. To clarify the hypothesis, PPR recording was performed and revealed that CCH impaired pre-synaptic neurotransmitter release. Furthermore, after blocking GABA function with bicuculline, we recorded a significantly increased PPR of MS–dCA1 circuits in 2VO rats, suggesting a decrease in pre-synaptic ACh release, which was further verified by decreased ChAT expression and ACh level in the dorsal hippocampus of 2VO rats. Interestingly, in our present study, we found that the application of GABA blocker failed to induce an increase of MS–dCA1 fEPSP in 2VO rats. Furthermore, the unchanged PPR after bicuculline administration suggested the same increased PPR of GABAergic MA-dCA1 circuit. These phenomena indicated the robust impairment of GABAergic transmission in the MS–dCA1 circuit of 2VO rats.

To further evaluate the effect of CCH on cholinergic post-synaptic function, we intracerebroventricularly injected scopolamine and mecamylamine to investigate the sensitivity of MS–dCA1 to cholinergic inhibitors. We found that the MS–dCA1 circuit of 2VO rats did not show a higher sensitivity to scopolamine but showed a higher sensitivity to mecamylamine. That is consistent with the previously reported nAChR reduction in the hippocampus (Lombardo and Maskos, 2015) but not consistent with the previously reported diminished mAChR in AD patients (Kihara and Shimohama, 2004; Scarr, 2012). Maybe it is because of the sequential impairment of pre-synaptic to post-synaptic function during AD progression (Hampel et al., 2018).

Transient forebrain ischemia following occlusion of the vertebral and common carotid arteries was reported inducing glutamate excitotoxicity and free radical formation (Gottlieb et al., 2006), and the hippocampal fEPSPs of CA3-CA1 circuit were found to be damaged due to impaired glutamatergic vesicle trafficking and vesicle release in CCH rat model (Yan et al., 2020; Zhang et al., 2020). These results suggested that impaired glutamatergic function might be the common feature in both acute and chronic brain ischemia. It has been known that, except for cholinergic and GABAergic projections, glutamatergic projection also contributes 7% of the connection between MS and the pyramidal cell layer of dCA1. However, whether glutamatergic projection is also impaired following CCH has not been identified. Besides, there is still a little amount of peri-somatic GABAergic interneurons in the pyramidal cell layer of dCA1 (Sun et al., 2014). Whether these conditions influence the results of our experiment still needs to be studied in the future.

In conclusion, 8-week CCH impairs the MS–dCA1 cholinergic-pyramidal cell circuits, including pre-synaptic neurotransmitter release and post-synaptic nAChR function. This will provide an important reference for understanding the neuropathological changes induced by CCH and for future drug research based on CCH rat model. However, the molecular mechanisms account for the CCH impaired pre-synaptic neurotransmitter release, and post-synaptic nAChR function still needs to be further studied.

## Data Availability Statement

The raw data supporting the conclusions of this article will be made available by the authors, without undue reservation.

## Ethics Statement

The animal study was reviewed and approved by Institutional Animal Care and Use Committee at Harbin Medical University.

## Author Contributions

JA and YX contributed to the conception and design of the project. YX, QS, X-QW, and Y-NC contributed to the conduct of the experiments and analysis of data. SZ wrote the manuscript. CM and SC helped with the manuscript revision. All authors contributed to the article and approved the submitted version.

## Conflict of Interest

The authors declare that the research was conducted in the absence of any commercial or financial relationships that could be construed as a potential conflict of interest.

## References

[B1] Ai J. Sun L. H. Che H. Zhang R. Zhang T. Z. Wu W. C.. (2013). MicroRNA-195 protects against dementia induced by chronic brain hypoperfusion *via* its anti-amyloidogenic effect in rats. J. Neurosci. 33, 3989–4001. 10.1523/jneurosci.1997-12.2013 23447608 PMC6619292

[B2] Alger B. E. Nagode D. A. Tang A. H. (2014). Muscarinic cholinergic receptors modulate inhibitory synaptic rhythms in hippocampus and neocortex. Front. Synaptic Neurosci. 6:18. 10.3389/fnsyn.2014.00018 25249974 PMC4155787

[B3] Ballinger E. C. Ananth M. Talmage D. A. Role L. W. (2016). Basal forebrain cholinergic circuits and signaling in cognition and cognitive decline. Neuron 91, 1199–1218. 10.1016/j.neuron.2016.09.006 27657448 PMC5036520

[B4] Bell L. A. Bell K. A. McQuiston A. R. (2013). Synaptic muscarinic response types in hippocampal CA1 interneurons depend on different levels of presynaptic activity and different muscarinic receptor subtypes. Neuropharmacology 73, 160–173. 10.1016/j.neuropharm.2013.05.026 23747570 PMC3783005

[B5] Bragin A. Jandó G. Nádasdy Z. Hetke J. Wise K. Buzsáki G. (1995). γ (40–100 Hz) oscillation in the hippocampus of the behaving rat. J. Neurosci. 15, 47–60. 10.1523/JNEUROSCI.15-01-00047.1995 7823151 PMC6578273

[B6] Chen X. Jiang X. M. Zhao L. J. Sun L. L. Yan M. L. Tian Y.. (2017). MicroRNA-195 prevents dendritic degeneration and neuron death in rats following chronic brain hypoperfusion. Cell Death Dis. 8:e2850. 10.1038/cddis.2017.243 28569780 PMC5520902

[B7] Cheng Q. Yakel J. L. (2014). Presynaptic α7 nicotinic acetylcholine receptors enhance hippocampal mossy fiber glutamatergic transmission *via* PKA activation. J. Neurosci. 34, 124–133. 10.1523/JNEUROSCI.2973-13.2014 24381273 PMC3866480

[B8] Cole S. L. Vassar R. (2009). Linking vascular disorders and Alzheimer’s disease: potential involvement of BACE1. Neurobiol. Aging 30, 1535–1544. 10.1016/j.neurobiolaging.2007.12.012 18289733 PMC3490488

[B9] Dannenberg H. Young K. Hasselmo M. (2017). Modulation of hippocampal circuits by muscarinic and nicotinic receptors. Front. Neural Circuits 11:102. 10.3389/fncir.2017.00102 29321728 PMC5733553

[B10] Deng M. Zhang Q. Wu Z. Ma T. He A. Zhang T.. (2020). Mossy cell synaptic dysfunction causes memory imprecision *via* miR-128 inhibition of STIM2 in Alzheimer’s disease mouse model. Aging Cell 19:e13144. 10.1111/acel.13144 32222058 PMC7253057

[B11] Du S. Q. Wang X. R. Xiao L. Y. Tu J. F. Zhu W. He T.. (2017). Molecular mechanisms of vascular dementia: what can be learned from animal models of chronic cerebral hypoperfusion? Mol. Neurobiol. 54, 3670–3682. 10.1007/s12035-016-9915-1 27206432

[B12] Dutar P. Bassant M. H. Senut M. C. Lamour Y. (1995). The septohippocampal pathway: structure and function of a central cholinergic system. Physiol. Rev. 75, 393–427. 10.1152/physrev.1995.75.2.393 7724668

[B13] Edwards R. H. (2007). The neurotransmitter cycle and quantal size. Neuron 55, 835–858. 10.1016/j.neuron.2007.09.001 17880890

[B14] ElAli A. Thériault P. Préfontaine P. Rivest S. (2013). Mild chronic cerebral hypoperfusion induces neurovascular dysfunction, triggering peripheral β-amyloid brain entry and aggregation. Acta Neuropathol. Commun. 1:75. 10.1186/2051-5960-1-75 24252187 PMC3843528

[B15] Ellis J. R. Ellis K. A. Bartholomeusz C. F. Harrison B. J. Wesnes K. A. Erskine F. F.. (2006). Muscarinic and nicotinic receptors synergistically modulate working memory and attention in humans. Int. J. Neuropsychopharmacol. 9, 175–189. 10.1017/s1461145705005407 15877932

[B16] Frerking M. Wilson M. (1996). Saturation of postsynaptic receptors at central synapses? Curr. Opin. Neurobiol. 6, 395–403. 10.1016/s0959-4388(96)80125-5 8794082

[B17] Gorelick P. B. Scuteri A. Black S. E. Decarli C. Greenberg S. M. Iadecola C.. (2011). Vascular contributions to cognitive impairment and dementia: a statement for healthcare professionals from the american heart association/american stroke association. Stroke 42, 2672–2713. 10.1161/strokeaha.111.634279 21778438 PMC3778669

[B18] Gottlieb M. Leal-Campanario R. Campos-Esparza M. Sánchez-Gómez M. Alberdi E. Arranz A.. (2006). Neuroprotection by two polyphenols following excitotoxicity and experimental ischemia. Neurobiol. Dis. 23, 374–386. 10.1016/j.nbd.2006.03.017 16806951

[B19] Hampel H. Mesulam M. Cuello A. Farlow M. Giacobini E. Grossberg G.. (2018). The cholinergic system in the pathophysiology and treatment of Alzheimer’s disease. Brain 141, 1917–1933. 10.1093/brain/awy132 29850777 PMC6022632

[B20] Hangya B. Borhegyi Z. Szilagyi N. Freund T. F. Varga V. (2009). GABAergic neurons of the medial septum lead the hippocampal network during theta activity. J. Neurosci. 29, 8094–8102. 10.1523/JNEUROSCI.5665-08.2009 19553449 PMC6666051

[B21] Hernández-Pérez J. Gutiérrez-Guzmán B. Olvera-Cortés M. (2016). Hippocampal strata theta oscillations change their frequency and coupling during spatial learning. Neuroscience 337, 224–241. 10.1016/j.neuroscience.2016.09.003 27615031

[B22] Honjo K. Black S. E. Verhoeff N. P. (2012). Alzheimer’s disease, cerebrovascular disease, and the β-amyloid cascade. Can. J. Neurol. Sci. 39, 712–728. 10.1017/s0317167100015547 23227576

[B23] Jedlicka P. Schwarzacher S. Winkels R. Kienzler F. Frotscher M. Bramham C.. (2009). Impairment of *in vivo* theta-burst long-term potentiation and network excitability in the dentate gyrus of synaptopodin-deficient mice lacking the spine apparatus and the cisternal organelle. Hippocampus 19, 130–140. 10.1002/hipo.20489 18767067

[B24] Kihara T. Shimohama S. (2004). Alzheimer’s disease and acetylcholine receptors. Acta Neurobiol. Exp. 64, 99–105. 15190684 10.55782/ane-2004-1495

[B25] Kim S. Nam Y. Jeong Y. O. Park H. H. Lee S. K. Shin S. J.. (2019). Topographical visualization of the reciprocal projection between the medial septum and the hippocampus in the 5×FAD mouse model of Alzheimer’s disease. Int. J. Mol. Sci. 20:3992. 10.3390/ijms20163992 31426329 PMC6721212

[B26] Levey A. I. Edmunds S. M. Koliatsos V. Wiley R. G. Heilman C. J. (1995). Expression of m1–m4 muscarinic acetylcholine receptor proteins in rat hippocampus and regulation by cholinergic innervation. J. Neurosci. 15, 4077–4092. 10.1523/JNEUROSCI.15-05-04077.1995 7751967 PMC6578239

[B27] Li Q. Yang C. Zhang X. Yang Z. Zhang T. (2019). Arginine vasopressin attenuates dysfunction of hippocampal theta and γ oscillations in chronic cerebral hypoperfusion *via* V1a receptor. Brain Res. Bull. 153, 84–92. 10.1016/j.brainresbull.2019.08.012 31430514

[B28] Lin J.-W. Faber D. S. (2002). Modulation of synaptic delay during synaptic plasticity. Trends Neurosci. 25, 449–455. 10.1016/s0166-2236(02)02212-9 12183205

[B30] Liu X. Tian L. Cui R. Ruan H. Li X. (2018). Muscarinic receptors in the nucleus accumbens shell play different roles in context-induced or morphine-challenged expression of behavioral sensitization in rats. Eur. J. Pharmacol. 819, 51–57. 10.1016/j.ejphar.2017.11.039 29196177

[B29] Liu C. D. Wang Q. Zong D. K. Pei S. C. Yan Y. Yan M. L.. (2016). Knockdown of microRNA-195 contributes to protein phosphatase-2A inactivation in rats with chronic brain hypoperfusion. Neurobiol. Aging 45, 76–87. 10.1016/j.neurobiolaging.2016.05.010 27459928

[B31] Liu X. J. Yuan L. Yang D. Han W. N. Li Q. S. Yang W.. (2013). Melatonin protects against amyloid-β-induced impairments of hippocampal LTP and spatial learning in rats. Synapse 67, 626–636. 10.1002/syn.21677 23620224

[B32] Lombardo S. Maskos U. (2015). Role of the nicotinic acetylcholine receptor in Alzheimer’s disease pathology and treatment. Neuropharmacology 96, 255–262. 10.1016/j.neuropharm.2014.11.018 25514383

[B33] Loreth D. Ozmen L. Revel F. G. Knoflach F. Wetzel P. Frotscher M.. (2012). Selective degeneration of septal and hippocampal GABAergic neurons in a mouse model of amyloidosis and tauopathy. Neurobiol. Dis. 47, 1–12. 10.1016/j.nbd.2012.03.011 22426397

[B34] Maalikjy Akkawi N. Borroni B. Agosti C. Magoni M. Broli M. Pezzini A.. (2005). Volume cerebral blood flow reduction in pre-clinical stage of Alzheimer disease: evidence from an ultrasonographic study. J. Neurol. 252, 559–563. 10.1007/s00415-005-0689-z 15726249

[B35] Müller C. Remy S. (2018). Septo-hippocampal interaction. Cell Tissue Res. 373, 565–575. 10.1007/s00441-017-2745-2 29250747 PMC6132648

[B36] Papke R. L. Sanberg P. R. Shytle R. D. (2001). Analysis of mecamylamine stereoisomers on human nicotinic receptor subtypes. J. Pharmacol. Exp. Ther. 297, 646–656. 10.1016/S1056-8719(01)00157-5 11303054

[B37] Raz L. Bhaskar K. Weaver J. Marini S. Zhang Q. Thompson J. F.. (2019). Hypoxia promotes tau hyperphosphorylation with associated neuropathology in vascular dysfunction. Neurobiol. Dis. 126, 124–136. 10.1016/j.nbd.2018.07.009 30010004 PMC6347559

[B38] Regehr W. G. (2012). Short-term presynaptic plasticity. Cold Spring Harb. Perspect. Biol. 4:a005702 10.1101/cshperspect.a005702 22751149 PMC3385958

[B39] Rubio S. E. Vega-Flores G. Martínez A. Bosch C. Pérez-Mediavilla A. del Río J.. (2012). Accelerated aging of the GABAergic septohippocampal pathway and decreased hippocampal rhythms in a mouse model of Alzheimer’s disease. FASEB J. 26, 4458–4467. 10.1096/fj.12-208413 22835830

[B40] Ruitenberg A. den Heijer T. Bakker S. L. van Swieten J. C. Koudstaal P. J. Hofman A.. (2005). Cerebral hypoperfusion and clinical onset of dementia: the Rotterdam Study. Ann. Neurol. 57, 789–794. 10.1002/ana.20493 15929050

[B41] Scarr E. (2012). Muscarinic receptors: their roles in disorders of the central nervous system and potential as therapeutic targets. CNS Neurosci. Ther. 18, 369–379. 10.1111/j.1755-5949.2011.00249.x 22070219 PMC6493542

[B42] Schmid L. C. Mittag M. Poll S. Steffen J. Wagner J. Geis H. R.. (2016). Dysfunction of somatostatin-positive interneurons associated with memory deficits in an Alzheimer’s disease model. Neuron 92, 114–125. 10.1016/j.neuron.2016.08.034 27641495

[B43] Schmitz T. W. Nathan Spreng R. Alzheimer’s Disease Neuroimaging Initiative (2016). Basal forebrain degeneration precedes and predicts the cortical spread of Alzheimer’s pathology. Nat. Commun. 7:13249. 10.1038/ncomms13249 27811848 PMC5097157

[B44] Solari N. Hangya B. (2018). Cholinergic modulation of spatial learning, memory and navigation. Eur. J. Neurosci. 48, 2199–2230. 10.1111/ejn.14089 30055067 PMC6174978

[B45] Sun L. H. Ban T. Liu C. D. Chen Q. X. Wang X. Yan M. L.. (2015). Activation of Cdk5/p25 and tau phosphorylation following chronic brain hypoperfusion in rats involves microRNA-195 down-regulation. J. Neurochem. 134, 1139–1151. 10.1111/jnc.13212 26118667

[B46] Sun Y. Nguyen A. Q. Nguyen J. P. Le L. Saur D. Choi J.. (2014). Cell-type-specific circuit connectivity of hippocampal CA1 revealed through Cre-dependent rabies tracing. Cell Rep. 7, 269–280. 10.1016/j.celrep.2014.02.030 24656815 PMC3998524

[B47] Teles-Grilo Ruivo L. M. Mellor J. R. (2013). Cholinergic modulation of hippocampal network function. Front. Synaptic Neurosci. 5:2. 10.3389/fnsyn.2013.00002 23908628 PMC3726829

[B48] Unal G. Joshi A. Viney T. J. Kis V. Somogyi P. (2015). Synaptic targets of medial septal projections in the hippocampus and extrahippocampal cortices of the mouse. J. Neurosci. 35, 15812–15826. 10.1523/JNEUROSCI.2639-15.2015 26631464 PMC4666911

[B49] Vandecasteele M. Varga V. Berenyi A. Papp E. Bartho P. Venance L.. (2014). Optogenetic activation of septal cholinergic neurons suppresses sharp wave ripples and enhances theta oscillations in the hippocampus. Proc. Natl. Acad. Sci. U S A 111, 13535–13540. 10.1073/pnas.1411233111 25197052 PMC4169920

[B50] Varga V. Hangya B. Kránitz K. Ludányi A. Zemankovics R. Katona I.. (2008). The presence of pacemaker HCN channels identifies theta rhythmic GABAergic neurons in the medial septum. J. Physiol. 586, 3893–3915. 10.1113/jphysiol.2008.155242 18565991 PMC2538919

[B51] Vega-Flores G. Rubio S. E. Jurado-Parras M. T. Gómez-Climent M. Á. Hampe C. S. Manto M.. (2014). The GABAergic septohippocampal pathway is directly involved in internal processes related to operant reward learning. Cereb. Cortex 24, 2093–2107. 10.1093/cercor/bht060 23479403 PMC4441070

[B52] Vilaró M. T. Palacios J. M. Mengod G. (1990). Localization of m5 muscarinic receptor mRNA in rat brain examined by *in situ* hybridization histochemistry. Neurosci. Lett. 114, 154–159. 10.1016/0304-3940(90)90064-g 2395528

[B53] Wess J. (2003). Novel insights into muscarinic acetylcholine receptor function using gene targeting technology. Trends Pharmacol. Sci. 24, 414–420. 10.1016/s0165-6147(03)00195-0 12915051

[B55] Yan M. L. Zhang S. Zhao H. M. Xia S. N. Jin Z. Xu Y.. (2020). MicroRNA-153 impairs presynaptic plasticity by blocking vesicle release following chronic brain hypoperfusion. Cell Commun. Signal. 18:57. 10.1186/s12964-020-00551-8 32252776 PMC7137307

[B56] Yang J. Hu Z. Jiang B. Ni L. Jin Y. Chen J.. (2011). Effect of chloramine-T on long-term potentiation at synapses between perforant path and dentate gyrus in hippocampus of rats *in vivo*. Neurotoxicology 32, 199–205. 10.1016/j.neuro.2011.01.006 21241739

[B57] Zamudio-Bulcock P. Valenzuela C. (2011). Pregnenolone sulfate increases glutamate release at neonatal climbing fiber-to-Purkinje cell synapses. Neuroscience 175, 24–36. 10.1016/j.neuroscience.2010.11.063 21130844 PMC3029476

[B58] Zhang H. Lin S. C. Nicolelis M. A. (2010). Spatiotemporal coupling between hippocampal acetylcholine release and theta oscillations *in vivo*. J. Neurosci. 30, 13431–13440. 10.1523/jneurosci.1144-10.2010 20926669 PMC2988451

[B59] Zhang S. Yan M. Yang L. An X. Zhao H. Xia S.. (2020). MicroRNA-153 impairs hippocampal synaptic vesicle trafficking *via* downregulation of synapsin I in rats following chronic cerebral hypoperfusion. Exp. Neurol. 332:113389. 10.1016/j.expneurol.2020.113389 32580014

